# Correction: Catalytic antibodies in arrhythmogenic cardiomyopathy patients cleave desmoglein 2 and N-cadherin and impair cardiomyocyte cohesion

**DOI:** 10.1007/s00018-023-04942-1

**Published:** 2023-09-20

**Authors:** Sunil Yeruva, Konstanze Stangner, Anna Jungwirth, Matthias Hiermaier, Maria Shoykhet, Daniela Kugelmann, Michael Hertl, Shohei Egami, Norito Ishii, Hiroshi Koga, Takashi Hashimoto, Michael Weis, Britt-Maria Beckmann, Ruth Biller, Dominik Schüttler, Stefan Kääb, Jens Waschke

**Affiliations:** 1 Chair of Vegetative Anatomy, Faculty of Medicine, Institute of Anatomy, Ludwig-Maximilians-University (LMU) Munich, Pettenkoferstrasse 11, 80336 Munich, Germany; 2 Department of Dermatology and Allergology, Philipps-University Marburg, 35037 Marburg, Germany; 3 Department of Dermatology, Keio University School of Medicine, Tokyo, Japan; 4 Department of Dermatology, Kurume University School of Medicine, Kurume, Japan; 5 Department of Dermatology, Graduate School of Medicine, Osaka City Metropolitan University, Osaka, Japan; 6 Krankenhaus Neuwittelsbach, Fachklinik Für Innere Medizin, Munich, Germany; 7 Department of Medicine I, University Hospital Munich, Ludwig-Maximilians University Munich (LMU), Campus Großhadern, Munich, Germany; 8 Institute of Legal Medicine, University Hospital Frankfurt, Goethe University, Frankfurt, Germany; 9 ARVC-Selbsthilfe E.V, Patient Association, Munich, Germany; 10 DZHK (German Centre for Cardiovascular Research), Munich Heart Alliance (MHA), Partner Site Munich, Munich, Germany; 11 Walter Brendel Centre of Experimental Medicine, Ludwig-Maximilians University Munich (LMU), Munich, Germany; 12 Interfaculty Center for Endocrine and Cardiovascular Disease Network Modelling and Clinical Transfer (ICON), LMU Munich, Munich, Germany; 13 Member of the European Reference Network for rare, low prevalance and complex diseases of the heart , ERN GUARD-Heart, Munich, Germany; 14 Present Address: Department of Otorhinolarynology, Technical University of Munich and University Hospital rechts der Isar, Ismaningerstrasse 22, 81675 Munich, Germany

**Correction: Cellular and Molecular Life Sciences (2023) 80:203** 10.1007/s00018-023-04853-1

In the published online article, the ESM files was not processed. The missed ESM has been processed in this article.

### Supplementary Information

Below is the link to the electronic supplementary material.

Supplementary file1 (DOCX 2374 KB)

